# Productivity and Quality of Alpine Grassland Vary With Soil Water Availability Under Experimental Warming

**DOI:** 10.3389/fpls.2018.01790

**Published:** 2018-12-14

**Authors:** Chengyang Li, Fei Peng, Xian Xue, Quangang You, Chimin Lai, Wenjuan Zhang, Yunxiang Cheng

**Affiliations:** ^1^Key Laboratory of Desert and Desertification, Northwest Institute of Eco-Environment and Resources, Chinese Academy of Sciences, Lanzhou, China; ^2^College of Resources and Environment, University of Chinese Academy of Sciences, Beijing, China; ^3^International Platform for Dryland Research and Education, Tottori, Japan; ^4^Arid Land Research Center, Tottori University, Tottori, Japan; ^5^College of Forestry, Fujian Agriculture and Forestry University, Fuzhou, China; ^6^State Key Laboratory of Grassland Agro-ecosystems, Key Laboratory of Grassland Livestock Industry Innovation, Ministry of Agriculture and Rural Affairs, College of Pastoral Agriculture Science and Technology, Lanzhou University, Lanzhou, China

**Keywords:** climate warming, community composition, importance value, biomass, forage quality, alpine meadow, desertification

## Abstract

The plant productivity of alpine meadow is predicted to generally increase under a warming climate, but it remains unclear whether the positive response rates will vary with soil water availability. Without consideration of the response of community composition and plant quality, livestock grazing under the current stocking rate might still lead to grassland degradation, even in meadows with high plant biomass. We have conducted a warming experiment from 2010 to 2017 to examine the interactive effects of warming and soil water availability on plant growth and forage quality at individual and functional group levels in an alpine meadow located in the permafrost region of the Qinghai–Tibetan Plateau. Warming-induced changes in community composition, biomass, and forage quality varied with soil water availability. Under dry conditions, experimental warming reduced the relative importance of grasses and the aboveground biomass by 32.37 g m^−2^ but increased the importance value of forbs. It also increased the crude fat by 0.68% and the crude protein by 3.19% at the end of summer but decreased the acid detergent fiber by 5.59% at the end of spring. The increase in crude fat and protein and the decrease in acid detergent fiber, but the decrease in aboveground biomass and increase the importance value of forbs, which may imply a deterioration of the grassland. Under wet conditions, warming increased aboveground biomass by 29.49 g m^−2^ at the end of spring and reduced acid detergent fiber by 8.09% at the end of summer. The importance value of grasses and forbs positively correlated with the acid detergent fiber and crude protein, respectively. Our results suggest that precipitation changes will determine whether climate warming will benefit rangelands on the Qinghai–Tibetan Plateau, with drier conditions suppressing grassland productivity, but wetter conditions increasing production while preserving forage quality.

## Introduction

The Qinghai–Tibetan Plateau (QTP) is the highest and largest plateau worldwide with a mean elevation of more than 4000 m above sea level (Wang et al., 2016). Alpine meadows cover approximately 7 × 10^5^ km^2^, accounting for about 50% of the total usable grassland on the QTP (You et al., 2017), and serve as the principal base of livestock husbandry for the local ethnic minority. However, alpine meadows have been severely degrading due to climate change and over-grazing activities (Chen et al., 2014; Wang et al., 2016). The warming on the QTP is predicted to be earlier and higher than other areas at the same latitude (Piao et al., 2004; Xu et al., 2014), and the livestock grazing intensity of the QTP has largely increased in the past 30 years (Song et al., 2009; Shang et al., 2014). For instance, the grassland area available per head of cattle declined from 13.73 ha in 1959 to 2.93 ha in 1999 in the Naqu region (Min and Cheng, 2001).

Climate warming will profoundly influence not only the above-ground biomass (AGB, Sullivan and Welker, 2005; Polley et al., 2013) but also the plant community composition and other ecosystem functions (Cardinale et al., 2012; Zhang et al., 2014). This can lead to changes in the nutritional status of individual species, and overall rangeland quality (Xu et al., 2018). For instance, an increase in legume abundance generally improves rangeland quality due to a high crude protein (g g^−1^, CP) in the legumes (Cantarel et al., 2013). Thus, the investigation of forage production, forage quality, and plant community composition in a changing climate, is fundamental for the sustainable use of the alpine meadow ecosystem.

Previous studies have reported increases (Wan et al., 2005; Sullivan et al., 2008; Lin et al., 2010; Li et al., 2011), decreases (De Boeck et al., 2007, 2008; Klein et al., 2007), and no change (Saleska et al., 2002; Liu et al., 2018) in AGB in response to a warmer climate. These different responses may be partly related to water availability, which has been shown to affect the warming impact on plant productivity and ecosystem carbon fluxes (Yang et al., 2009; Peng et al., 2015). For example, in cold and humid ecosystems with abundant soil water, warming generally has positive effects on plant growth directly by stimulating temperature-driven physiological processes and indirectly by extending the growing season length (Wan et al., 2005; Liu et al., 2018). In contrast, in high-elevation ecosystems that face water shortage, warming will cause further water limitation for the carbon uptake process, and negatively influence plant growth (Xue et al., 2009; Chelli et al., 2017).

Plant community composition can mediate the response of AGB and forage quality to warming and the warming-induced change in soil moisture because of the different traits of different plant types. For example, grasses are more affected by drought than forbs in alpine systems because they have a low relative reduction in stomatal conductance (Bolling and Feller, 2014; Wellstein et al., 2017; Ganjurjav et al., 2018). Forbs have high root biomass, and superior interspecific competition ability, which could make them better able to cope with a warmer and drier climate (Dunnett and Grime, 1999; Klein et al., 2007; Zhang et al., 2014). Any increases in forage production may be compromised by a decrease in forage quality via nutrient-dilution effects (Shi et al., 2013). For example, a meta-analysis for grassland showed an increase in structural carbohydrates and lignification even though the forage production was enhanced in a warming climate (Dumont et al., 2015). CP, crude fat (EE), and acid detergent fiber (ADF) are indicators that can be used to reflect the forage quality. Pastures with higher CP and EE have higher nutritional value, while the higher the ADF content in pastures, the poorer the nutritional value of the pasture (Deak et al., 2007; Shi et al., 2013; Xu et al., 2018).

Although rangeland quality has been investigated in terms of decline of medicinal and non-palatable forbs (Klein et al., 2007), to our knowledge, no studies have examined rangeland quality at the community level. From previous studies in the same ecosystem, we know that the duration of soil-thaw period has extended (Xue et al., 2014), which suggests a long growing season, and the high availability of soil nitrogen in the warming treatment (Peng et al., 2016). However, the warming effect on ecosystem carbon fluxes and AGB varies with the annual rainfall amount (Peng et al., 2014). The AGB increased only when the annual rainfall was much higher than the long-term average. The gross ecosystem production and ecosystem respiration positively relate to the soil moisture when it is less than ∼15% but negatively relate to soil moisture when it is higher than the threshold (Peng et al., 2014). Warming will decrease surface layer soil moisture (Xue et al., 2014) because of elevated evapotranspiration (Peng et al., 2015). We expect warming to lead to a decrease of grass species but increase in forb species in localities where soil moisture is low. Thus, we hypothesize that a warmer climate would stimulate growth, leading to improved forage quality where local soil water availability is high. Conversely, growth would be inhibited in drier areas, leading to poorer forage quality.

## Materials and Methods

### Site Description

**FIGURE 1:**
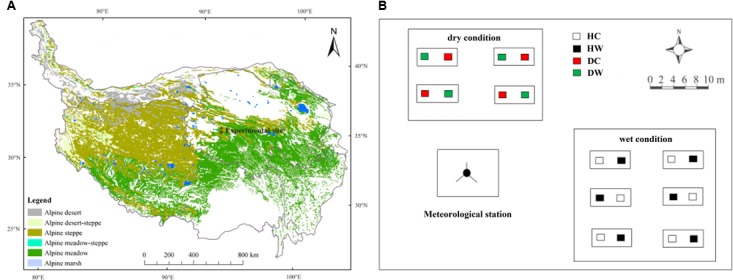
The location of the research site **(A)**, and layout of the experimental plots **(B)**. HC and HW represent control and warmed under wet conditions, DC and DW represent control and warmed under dry conditions.

The experimental site is located near the QTP Research Base of the State Key Laboratory of Permafrost Engineering, Chinese Academy of Sciences (34°49′34″–34°49′37″N, 92°55′57″–92°56′06″E), with a mean elevation of 4635 m above sea level (Figure 1A). Based on meteorological station data (daily observations) collected from January 2010 to December 2013, the mean annual temperature is −3.8°C, with monthly air temperature ranging from −27.9°C in January to 19.2°C in July. Mean annual precipitation is 290.9 mm, over 95% of which falls from May to October. Mean potential annual evaporation is 1316.9 mm, mean annual relative humidity is 57% and mean annual wind velocity is 4.1 m s^−1^ (Xu et al., 2015). The frozen period lasts from September to April and the depth of the seasonally frozen soil generally ranges from 2 to 3 m (Wang et al., 2007). The study site is a summer grazed range, dominated by alpine meadow vegetation, such as *Kobresia pygmaea* (sedge), *Kobresia capillifolia* (sedge), and *Carex moorcroftii* (sedge). *Polygonum viviparum* (forb) and *Stipa purpurea* (grass) are also common. The mean plant height is about 5 cm at a community level. Plant roots are mainly within the 0–20 cm soil layer. Soil development is weak, and soils are classified as Mattic Cryic Cambisols (Alpine meadow soil, as Cambisols in FAO/UNESCO taxonomy) with a mattic epipedon at a depth of approximately 0–10 cm and an organic-rich layer at 20–30 cm (Wang et al., 2007). The experimental field was on a mountain slope with a mean inclination of 5°.

### Experimental Design

Experimental plots were established in a typical alpine meadow within about 300 m of the Research Base. The experimental setup was finished in June 2010 and the plots were protected by a fence with hard iron wires to keep large herbivores away. A completely randomized split-plot experiment design was used (Figure 1B). Detailed information about soil properties in the 0–20 cm layer, soil temperature and soil moisture in the 0–10 cm layer, and plant features under dry and wet conditions are presented in Supplementary Table S1. Species composition was similar throughout the experimental plots but with lower coverage and plant height under dry than wet conditions. The average elevation difference between plots under dry and wet conditions was about 1 m.

In the experiment, the study site was split into large blocks with dry (6.55% annual mean in the 0–10 cm layer, v/v%) and wet (11.08%, v/v%) soils. Each block was split into small sub-plots in which warming treatments were applied. In the dry block, there were four pairs of control (DC) and warmed plots (DW); in the wet block, there were six pairs of control (HC) and warmed (HW), which represents four and six replicates under dry and wet conditions, respectively. There were 20 plots in total. Each plot occupied a 2 m × 2 m area. In each warmed plot, one 165 cm × 15 cm infrared heater (MR-2420, Kalglo Electronics Inc., Utah, United States) was suspended in the middle of the plot at 1.5 m above the ground with a radiation output of 150 W m^−2^. Compared with the control, warming increased soil temperature by 1.8°C at 10 cm soil depth (Xue et al., 2014). The heating worked yearly-round since July 1st, 2010 and continued until October 1st, 2017. Each control plot had a “dummy” radiator (no heating element) with the same dimensions as the infrared radiators suspended at a similar height to rule out any effects of shading by heaters (Xue et al., 2011). Moreover, in an open and flat area, a permanent meteorological observation station of 4 m height was installed to automatically record the climate variables outside of the experimental plots.

### Temperature and Moisture Measurements

A thermo-probe (Model 109, Campbell Scientific, Inc, Utah, United States) was installed at the 15 and 30 cm soil depth in the center of each plot to monitor the soil temperature. Volumetric soil water content (v/v%) was measured by frequency domain reflectometry (FDR, EnviroSmart sensor, Sentek Pty Ltd., Stepney, Australia) at depths of 0–10, 10–20, and 20–40 cm. The daily average soil temperature and moisture were recorded with a CR 1000 data logger (Campbell Scientific, Inc., Utah, United States) at 10-min intervals. The 10-min recorded data were then averaged into daily data. The warming effects on soil temperature and soil moisture were analyzed according to the daily data. Although the warming experiment started in 2010, the species composition was investigated in June and September 2017. Thus, the warming impact on soil moisture was reported based on data for 2017. Soil temperature at 15 and 30 cm soil depth in the experimental sites was also reported based on data in 2017.

### Vegetation Characteristic Measurements

In the growing season of 2017 (early June and late September), the vegetation characteristics of each plot were determined by the following methods. The experimental field (2 m × 2 m) was divided equally into four parts diagonally. Ten individuals were measured for height randomly in each part. The coverage of each part was measured with a frame with the interior dimensions of 27 cm × 27 cm. The heights and coverage from the four parts were averaged for the height and coverage of each plot. The AGB was measured by cutting all the visible individuals above the ground (30 cm × 30 cm) in the center of each plot. Each species was identified then put in different envelopes. The collected biomass was then air dried, and then sent back to the laboratory and put into an oven to dry for 48 h at 75°C.

The height and frequency of each species were measured in a 20 cm × 20 cm small subplot within each plot. A frame with 100 small quadrants (1 cm × 1 cm) was used to measure the frequency. The height of each species was assessed based on the number of individuals for the species. If the number of individuals of a given species was less than 20, the height of the species was averaged by the number of heights measured. Otherwise, the height of that species was derived by 20 measurements with a ruler. The measured heights were then averaged for each species. Changes in importance values (IV) of species can reflect the variation of plant community composition (Zhang et al., 2014; Xu et al., 2015; Peng et al., 2017). The IV was initially developed to investigate the community composition and structure for forestry studies. The parameters used to calculate this index are density, basal area of each tree, and the frequency of a specific species. In the case of herbaceous vegetation, IV should be calculated based on the aboveground biomass. The height, coverage, and frequency are the most important factors to determine the biomass of each species of the herbaceous plants. Thus, we used the modified version of IV to characterize the community. The IV of each species was derived from relative coverage, relative height, and relative frequency. The relative height of a species is the ratio of the average height of that species to the summed height of all the species in the plot. The IV of each plant functional group was the sum of the IVs of any species belonging to that group.

$${\mathrm{IV}}_{i}=(\mathrm{rc}+\mathrm{rh}+\mathrm{rf})/3$$

where *IV_i_* is the importance value of a specific species, *rc* is the relative coverage of species, *rh* is the relative height of species, and *rf* is the relative frequency of species.

The root biomass was sampled with a soil corer (7 cm internal diameter). In the 2016 growing season (late August), the soil with roots was extracted every 10 cm to a depth of 50 cm in each plot. The soil cores were placed in the cooler immediately and then transported to the laboratory by train. In the laboratory, soil samples were air-dried and crumbled by hand to pass through a 2-mm diameter sieve to remove large particles from the finer soil in distilled water. Then, the fine living roots were hand-picked based on their color and consistency in a distilled water bath, and the separated roots were dried at 75°C for 48 h.

### Forage Quality Measurement

Plant species were classified into the three functional groups: grasses, sedges, and forbs. Only one legume species (*Astragalus polycladus*) was found in the study site and it was rare, therefore, we grouped it with forbs. Because of the lower statue of alpine plants and the need to avoid the interactive effect of warming and clipping, we only collected aboveground biomass for quality measurement in a 20 cm × 20 cm subplot, therefore, the biomass was not enough to measure the CP, ADF, and EE of each functional group. As a result, the CP, ADF, and EE of the AGB mixture were measured for each plot. The CP was determined with an Automatic Kjeldahl Nitrogen Determination Apparatus (Kjeltec 8100, FOSS, Höganäs, Sweden). The ADF was determined by a sequential detergent fiber analysis (Goering and Van Soest, 1970), and the EE was determined by the Soxhlet extraction method (ANKOM XT15i, United States).

### Data Analysis

A split-plot analysis of variance (ANOVA) was used to examine the main and interactive effects of warming and soil moisture conditions on the community AGB; belowground biomass; AGB of individual species; plant coverage; the IV of grasses, sedges, and forbs; and CP, ADF, and EE. One-way ANOVA was used to examine the effect of warming under dry and wet conditions on soil moisture; soil temperature; AGB; belowground biomass; AGB of individual species; plant coverage; individual species coverage; the IV of grasses, sedges, and forbs; and CP, ADF, and EE. Simple linear regression analyses were conducted to examine the correlation between the IV of grasses and ADF, the IV of forbs, and CP. All the above-mentioned statistical analyses were conducted using SPSS.17.0 for Windows (SPSS, Inc., Chicago, IL, United States).

## Results

### Microclimate

**FIGURE 2:**
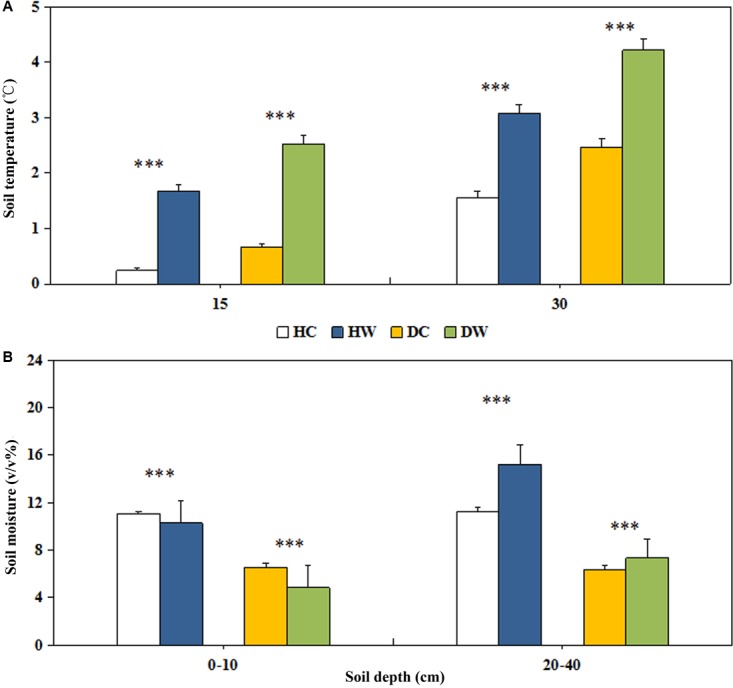
Effects of warming on soil temperature at the depth of 15cm and 30cm **(A)**, soil moisture at the depth of 0–10 cm and 20–40 cm **(B)** under wet and dry conditions. See Figure 1 for meanings of abbreviations. The error bars indicate standard error. Significance: ^∗∗∗^*P* < 0.001.

The mean annual soil temperature was lower under wet conditions (15 cm, 0.96°C; 30 cm, 2.32°C) than dry conditions (15 cm, 1.60°C; 30 cm, 3.35°C; Figure 2A). Experimental warming significantly increased the soil temperature by 1.43 and 1.52°C at 15 and 30 cm soil depth, respectively, in the HW compared with the HC plots. It increased the soil temperature by 1.87 and 1.85°C at 15 and 30 cm soil depth, respectively, in the DW compared with DC plots (*P* < 0.001; Figure 2A). Soil water content was lower under dry conditions (0–10 cm, 6%, v/v%; 20–40 cm, 7%, v/v%) than wet conditions (0–10 cm, 11%, v/v%; 20–40 cm, 13%, v/v%) (Figure 2B). These values represent a significant decrease of 1% and 2% (v/v%; *P* < 0.001) in HW and DW plots in 0–10 cm layer, respectively, and increase by 4% and 1% (v/v%; *P* < 0.001) in HW and DW plots in the 20–40 cm layer, respectively (Figure 2B).

### Biomass and Plant Coverage

**FIGURE 3:**
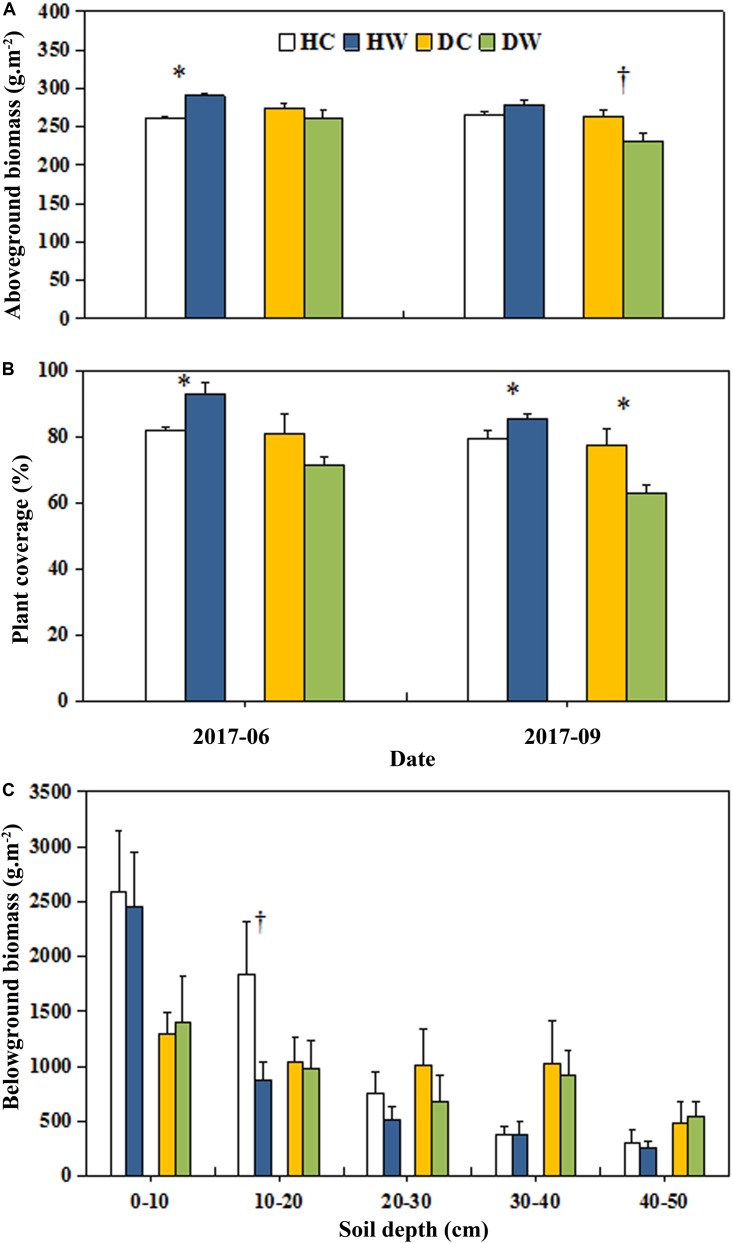
Effects of warming on AGB **(A)**, plant coverage **(B)**, and belowground biomass **(C)** under wet and dry conditions. See Figure 1 for meanings of abbreviations. The error bars indicate standard error. Significance: ^†^*P* < 0.1; ^∗^*P* < 0.05.

**FIGURE 4:**
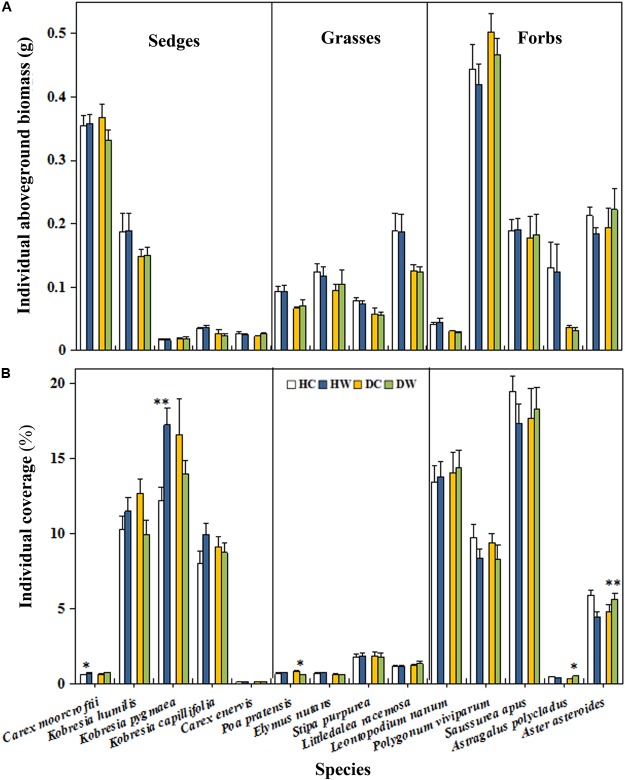
Effects of warming on biomass **(A)** and coverage **(B)** of individual species under wet and dry conditions. See Figure 1 for meanings of abbreviations. The error bars indicate standard error. Significance: ^†^*P* < 0.1; ^∗^*P* < 0.05; ^∗∗^*P* < 0.01.

Warming had no overall effect on the total AGB and plant coverage of a plot (Table 1). However, the interaction between warming and measuring month had a significant effect on the total AGB (*P* < 0.05). A significant interaction between warming and soil moisture condition on the plant coverage was observed (*P* < 0.001; Table 1). The plant coverage and AGB of a plot decreased by 15% (*P* < 0.05) and 32.37 g m^−2^ (*P* < 0.1) in September in the DW plots but they increased by 11% (*P* < 0.05) and 29.49 g m^−2^ (*P* < 0.05) in June in the HW plots (Figures 3A,B). Neither warming nor the interaction of warming with soil moisture condition affected the AGB of each individual species (Table 1 and Figure 4A) Warming significantly increased the coverage of *C. moorcroftii* by 0.12% in the HW plots (sedge; *P* < 0.05, Table 1) and *Aster asteroides* by 0.86% in the DW plots (forb; *P* < 0.01, Table 1). However, it decreased the coverage of *Poa pratensis* by 0.24% (*P* < 0.01) in the DW plots (Figure 4B). The interaction between warming and soil moisture condition had a significant effect on the coverage of *K. humilis* (sedge; *P* < 0.1), *K. pygmaea* (sedge; *P* < 0.01) and *A. polycladus* (forb; *P* < 0.05, Table 1). The coverage of *K. humilis* decreased by 2.74% (*P* < 0.1) but that of *A. polycladus* increased by 0.18% (*P* < 0.05) in the DW plots and the coverage of *K. pygmaea* increased by 5.06% (*P* < 0.01) in the HW plots (Figure 4B).

**Table 1:** Results (*F*-values) of split-plot ANOVA analysis of the effect of soil moisture condition (P), warming treatment (W), and their interactions on aboveground biomass (AGB), plot average plant coverage (PC), below-ground biomass (BGB), individual aboveground biomass (IAGB) of *Carex moorcroftii* (C.m), *Kobresia humilis* (K.h), *Kobresia pygmaea* (K.p), *Aster asteroides* (A.a), and *Astragalus polycladus* (A.P).

Variance source	AGB	BGB	PC	IAGB	C.m	K.h	K.p	A.a	A.p
P	5.47^∗^	0.79	29.42^∗∗∗^	8.94^∗∗^	0.80	0.21	0.16	0.00	0.19
W	0.03	1.54	0.82	0.40	7.13^∗^	0.64	0.82	9.43^∗∗^	1.25
P^∗^W	1.70	0.62	22.63^∗∗∗^	0.03	0.29	4.32^†^	7.91^∗^	0.67	6.18^∗^

*Significance: ^†^p < 0.1; ^∗^p < 0.05; ^∗∗^p < 0.01; ^∗∗∗^p < 0.001.*

Neither warming nor the interaction of warming with soil moisture condition had any significant effect on the total belowground biomass (Table 1). Belowground biomass decreased by 970 g m^−2^ (*P* < 0.1) at 10–20 cm soil depth in the HW plots but no change in the DW plots (Figure 3C). The proportion of the belowground biomass at 0–20 cm soil depth was lower but it was higher at the depth of 30–50 cm under dry conditions (the average belowground biomass of DC and DW plots) than wet conditions (the average belowground biomass of HC and HW plots, Figure 3C).

### Plant Community Composition

**FIGURE 5:**
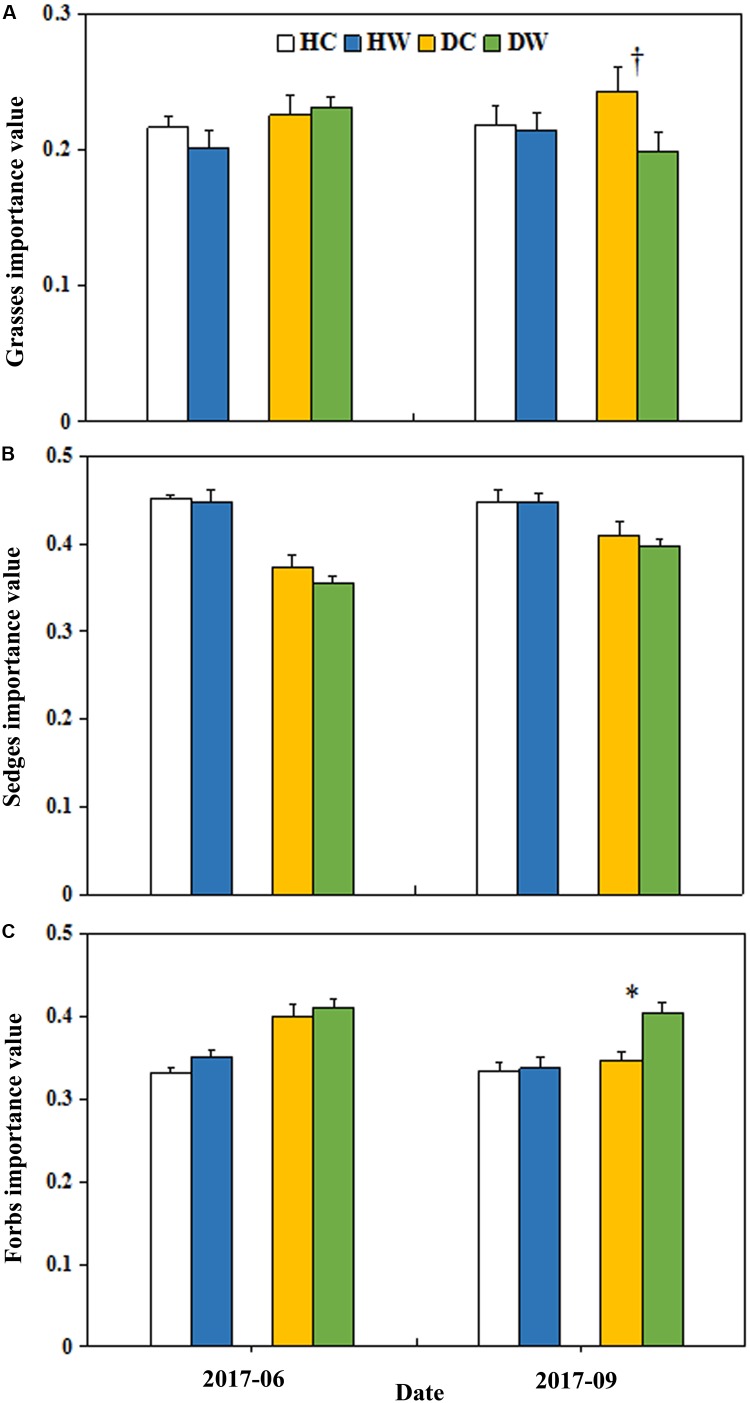
Effects of warming on the IV of grasses **(A)**, sedges **(B)**, and forbs **(C)** under wet and dry conditions. See Figure 1 for meanings of abbreviations. The error bars indicate standard error. Significance: ^∗∗∗^*P* < 0.1; ^∗^*P* < 0.05.

Warming significantly decreased the total IV of grasses (*P* < 0.05; Table 2). The interaction between warming, month, and soil moisture condition had a significant effect on the IV of grasses (*P* < 0.05) but no significant effect on the IV of sedge. The average IV of grasses was lower in DW plots (0.20) compared with DC plots (0.24, *P* < 0.1) in September 2017 (Figure 5A). The average IV of sedges under dry conditions was lower than wet conditions (Figure 5B), but the average IV of forbs reversed (Figure 5C). Warming increased the total IV of forbs (*P* < 0.1; Table 2). The interaction of warming with soil moisture condition had no significant effect on the IV of forbs (Table 2). The IV of forbs increased by 0.05 in DW plots (0.40) compared with the DC plots (0.35, *P* < 0.05) in September but there was no change in the HW plots (Figure 5C).

**Table 2:** Results (*F*-values) of split-plot ANOVA analysis of the effect of soil moisture condition (P), warming treatment (W), and their interactions on ADF, CP, EE, and importance value of different functional groups (IV).

Variance source	ADF	CP	EE	Grasses IV	Sedges IV	Forbs IV
P	10.08^∗∗^	5.30^∗^	6.56^∗^	5.63^∗^	39.17^∗∗∗^	26.36^∗∗∗^
W	10.85^∗∗^	0.20	0.00	5.51^∗^	0.44	3.91^∗∗∗^
P^∗^W	0.01	10.18^∗∗^	7.74^∗∗^	1.49	0.00	0.47

*Significance: ^∗∗∗^p < 0.1; ^∗^p < 0.05; ^∗∗^p < 0.01; ^∗∗∗^p < 0.001.*

### Forage Quality

**FIGURE 6:**
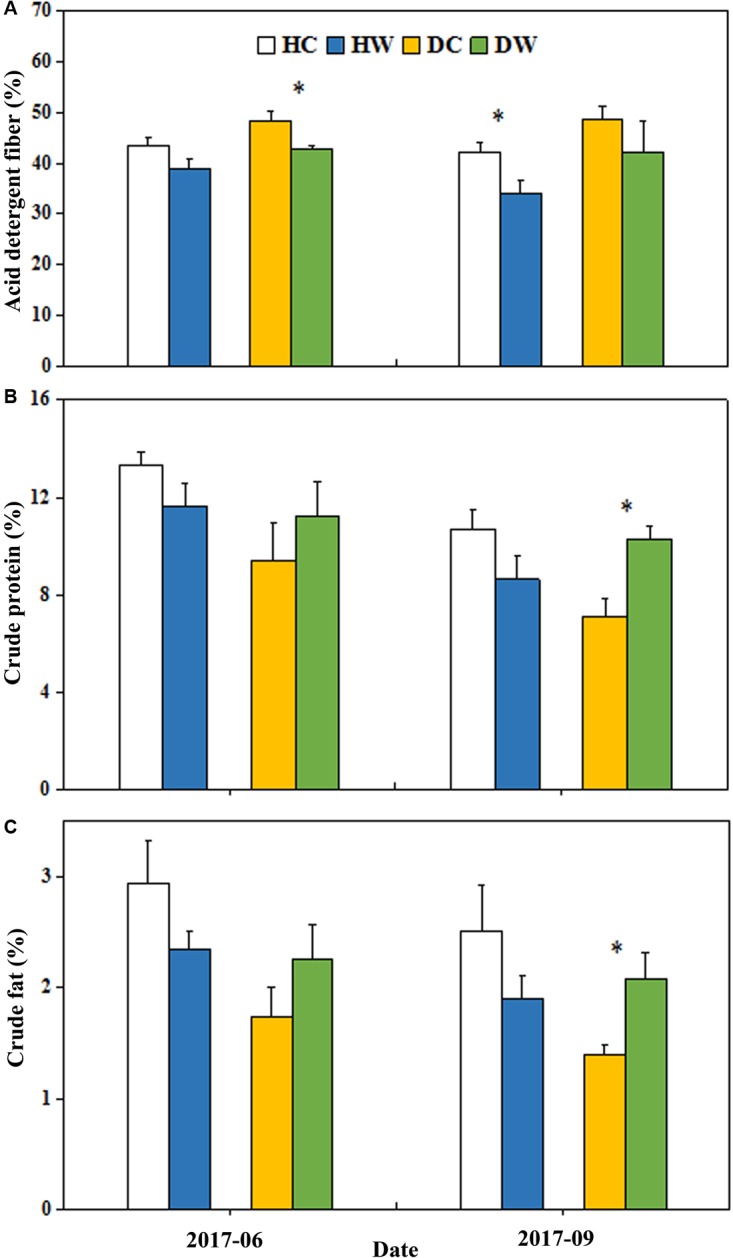
Effects of warming on ADF **(A)**, CP **(B)**, and EE **(C)** under wet and dry conditions. See Figure 1 for meanings of abbreviations. The error bars indicate standard error. Significance: ^∗^*P* < 0.05.

Warming significantly reduced the ADF (*P* < 0.01; Table 2). No interactive effects of warming with soil moisture condition were observed on ADF (Table 2). The ADF decreased in June by 5% in the DW plots (48%) compared with the DC plots (43%, *P* < 0.05) and it reduced only in September by 8% in the HW plots (42%) relative to the HC plots (34%, *P* < 0.05, Figure 6A). Warming had no significant effect on the total CP (Table 2). The interaction between warming and soil moisture condition significantly affected the CP (*P* < 0.01, Table 2), which increased by 3% in the DW plots (10%) compared with the DC plots (7%, *P* < 0.05) in September 2017 (Figure 6B). Warming had no significant effect on the total EE (Table 2). The interaction between warming and soil moisture condition had a significant effect on EE (*P* < 0.01, Table 2), which was 1% higher in the DW plots (2%) relative to the DC plots (1%, *P* < 0.05) in September (Figure 6C).

### Relationship Between Forage Quality and Importance Value of Different Groups

**FIGURE 7:**
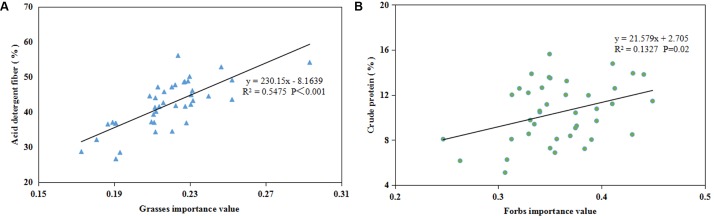
The correlation between the IV of grasses and ADF **(A)**, the IV of forbs and CP **(B)**.

The pooled data showed a positive correlation between the ADF and the IV of grasses in the control and warming plots (*P* < 0.001, Figure 7A), and a positive correlation between the CP and the IV of forbs (*P* = 0.02, Figure 7B).

## Discussion

### Response of Soil Moisture to Warming

The hydrological cycle in the ecosystem is driven by energy transfer. Soil surface evaporation and plant transpiration always accompany heat transfer from the soil to the atmosphere. In our study system, experimental warming increased soil surface temperatures, and temperature gradients between the soil surface and the atmosphere (Xue et al., 2014). This led to a higher evaporation and a reduction in soil water in the upper layers (Figure 2B), where most of the plant roots were located (0–20 cm, Figure 3C). Warming may foster a less favorable belowground environment for plant species with respect to soil temperature and water content. The study area is located in an area with continuous permafrost and the thickest active layer in China (Xue et al., 2014; Peng et al., 2017). Experimental warming enhanced the thawing process in the active layer during the warming season (Xue et al., 2014). Longer thaw duration and the higher active layer thickness might be responsible for increased soil water content in the deeper soil layers (Figure 2B).

### Response of Biomass to Warming

An increase in temperature could stimulate plant growth, but the associated soil moisture decline might offset the positive effect of increased temperature on plant biomass (De Boeck et al., 2008, Chelli et al., 2016, Liu et al., 2018). This might explain the non-detectable overall effect on AGB as soil moisture declined in our study (Figure 2B). Warming decreased the AGB in end of summer under dry conditions and increased the AGB at the end of spring under wet conditions (Figure 3A). This confirmed the hypothesis that a warmer climate would stimulate growth where local soil water availability is high, while growth would be inhibited in drier areas. Although soil moisture declined at 0–10 cm soil depth under both dry and wet conditions, the soil moisture in HW plots was higher than that in DC plots (Figure 2B). The reduced soil moisture might not limit the plant growth under wet conditions but constrains it under dry conditions. We did not directly measure the growing season duration; however, the advanced thawing of the active layer in spring (Xue et al., 2014) indicates an early start of the growing season in the permafrost region, thus resulting in the enhanced AGB in end of spring under wet conditions. Even the freezing of active layer delays (Xue et al., 2014) plants cannot make use of the extended period of thermally favorable conditions, probably because of an established life cycle (Yu et al., 2010) due to the low temperature (Dong et al., 2009). The long-term evolution and adaptation of alpine plants cause them to have low heat requirements even in the summer developmental stages (Yu et al., 2010). Warming in summer thus rapidly accelerates plant development, leading to earlier completion of the reproductive cycle of species (Yu et al., 2010). The possible early end of growing period under the high-magnitude soil temperature increase, under dry conditions (Figure 2A) might explain the decline in AGB in the DW plots at the end of summer.

Given no change in the individual aboveground biomass of the species in either the DW or HW plots (Table 1 and Figure 4C), the enhancement in AGB under wet conditions and reduction under dry conditions can be partly ascribed to the changes in community composition (Zhang et al., 2014; Xu et al., 2018). In general, the height of grass species is greater than that of forb species. The decrease in the IV of grasses due to the reduction in plant height and coverage of grasses (Figures 5A,C) in our study might partly bring about a decrease in AGB under dry conditions. Under wet conditions, the relatively higher soil moisture (Figure 2B) might be adequate to offset the negative effects of surface drying after warming, and the elongation of the growing season could support the increased AGB in wet locations.

The warming-induced decrease in belowground biomass is mainly due to drought stress in the surface layer (De Boeck et al., 2007; Klein et al., 2007). Plant belowground biomass was mostly distributed in upper soil layers in our experimental sites (Figure 3C), which might explain the decrease in belowground biomass at 0–20 cm soil depth in the HW plots. Forb species have deep roots (Peng et al., 2017). The higher IV of forbs under dry than wet conditions (Figure 5C), thus may be responsible for the higher proportion of the belowground biomass at 30–50 cm soil depth under dry than wet locations (Figure 3C).

### Response of the Community Composition to Warming

The abundance of different plant functional groups responds distinctly to climate warming depending on the traits that can enhance survival. For example, the abundance of grasses decreased with the presence of forbs under dry conditions (Dunnett and Grime, 1999) because forbs are more adaptable to climate warming than grasses species because of their high interspecific competitive ability for resources (Dunnett and Grime, 1999; Klein et al., 2007; Klanderud, 2008). The competitive advantage of forbs (Klein et al., 2007) could therefore result in the decrease in IV of grasses but the increase in IV of forbs in the DW plots (Figure 5C). This confirms the hypothesis that warming could lead to a decrease in grass species but increase in forb species in areas of low soil moisture.

The responses of different plant functional types could vary with precipitation or nutrient availability due to the niche difference or complementary use of moisture and nutrients (Peng et al., 2017; Xu et al., 2018). For example, the frequency and abundance of grasses decreased in the relatively dry sites in a sub-alpine meadow warming study (Rudgers et al., 2014) but that of sedge and grasses increased in a wet tundra (Elmendorf et al., 2012). The soil water conditions in the sub-alpine meadow and the wet tundra may explain the opposite responses of grasses and sedge abundance to warming. Warming can aggravate soil drought stress (Xue et al., 2017) on plant growth under dry sites, which likely causes plants to distribute more biomass belowground to obtain more nutrients (Zhang et al., 2014). The competitive disadvantage of grasses leads to the reduction in IV of grass species (Wang et al., 2010; Xu et al., 2014). Enhancement of water uptake capacity of forbs under drought stress was reported in an alpine ecosystem on the QTP (Ganjurjav et al., 2018), whereas forbs are less resistant to drought than grasses in alpine grassland with an elevation less than 3000 m (De Boeck et al., 2016, 2018).

In our study system, we found a higher average IV of forbs under dry than wet conditions (Figure 5C), which suggests the dependence of variation in response of plant groups to warming on the site conditions. In alpine ecosystems, under wet conditions, adequate moisture can satisfy the growth of grasses and forbs, therefore weakening the inter-specific competition (Wang et al., 2010; Zhang et al., 2014). This could partly support the non-significant change in the IV of grasses and forbs under wet conditions (Figures 5A,C). One study carried out in the alpine grassland ecosystems on the eastern QTP found that both experimental warming and drought treatments increased grass abundance and decreased sedge and forb abundance (Liu et al., 2018). The difference between our results and those of Liu et al. might be attributed as follows first, different community composition in two study sites. In Liu et al.’s study, grasses are the dominant functional groups, the grass abundance (about 0.6) was significantly higher than that of forbs (about 0.3) and sedges (about 0.1). However, there were fewer grasses in our study site, the IV of forbs (about 0.4) and sedges (about 0.4) was significantly higher than that of grasses (about 0.2). Warming is expected to increase biotic interactions and competition among species under dry conditions (Olsen and Klanderud, 2014). Dominant species generally have a more competitive position than other functional groups (Dunnett and Grime, 1999; Klanderud, 2008). Second, in Liu’s study, mean annual precipitation is 489 mm. Grass abundance is highly significantly correlated with water condition across the altitude gradient on the QTP (Dorji et al., 2014) and decreases with the presence of forbs under dry conditions (Dunnett and Grime, 1999). This suggests a threshold after which graminoids are no longer able to compete with forbs if precipitation decreases further in alpine systems where few grasses exist compared with forbs. The low mean annual precipitation (290.9 mm) in our study area might also be the cause of the reduction in IV of grasses and increased in IV of forbs under dry conditions.

### Response of Forage Quality to Warming

Rangeland quality was determined by community nutrient production, which included two aspects: forage production (AGB) and forage quality (Xu et al., 2018). Forage production provides edible fodder to livestock and directly affects grassland carrying capacity, while forage quality determines the efficiency of pasture utilization and affects livestock growth (Shi et al., 2013). Shifts in plant community structure caused by warming could have further effects on forage quality (Dumont et al., 2015). Grass species contains more ADF than other plant functional groups (Xu et al., 2018). In general, the higher the EE and nitrogen-free extracts, especially the CP in the pasture, the better the nutritional value, and the higher the ADF content, the poorer the pasture’s nutritional value (Shi et al., 2013). In general, CP and ADF are positively and negatively correlated with livestock productivity, respectively (Lee et al., 2016). In our experiment, warming reduced the ADF content both under dry and wet conditions but increased the CP content under dry conditions. The positive correlation between ADF and IV of grasses (Figure 7A) and the decrease in IV of grasses indicate the change in grass abundance may have a positive effect on the forage quality. Alpine plants have a series of adaptive mechanisms in response to warming and drought (Shi et al., 2013), such as decreasing structural-carbohydrate content and increasing CP content to reduce damage caused to the plant by warming and drought (Shi et al., 2013; Xu et al., 2018). Results on the northeastern QTP show that legumes have the highest nutritive value, followed by non-legume forbs, sedges, and grasses according to the nutrient content ranking (Xu et al., 2018), which supports our observation of the positive correlation between ADF and IV of grasses. The positive correlation between CP and the IV of forbs (Figure 7B) could lead to the CP increase as IV of forbs increased in the DW plots at the end of summer (Figure 6B). The negative correlation between EE and precipitation (Shi et al., 2013) could support the EE increase in the DW plots.

In our study, warming improved forage quality due to reduced ADF content and increased EE and CP content under dry conditions. However, warming had no significant effect on the CP and EE under wet conditions, which was not as we expected. The forb increase may contribute to improved forage quality due to the higher CP content of forbs than grasses. No change in community composition may result in no significant response of CP content to warming under wet conditions. Warming may improve forage quality owing to stimulation of legume growth on the northeastern QTP (Wang et al., 2012; Xu et al., 2018). In our experimental site, there was only one legume species, and it was rare. Klein et al. (2007) showed that warming reduced rangeland quality due to increased production of the non-palatable forb *Stellera chamaejasme* and the decreased production of the medicinal forb *Gentiana straminea*. However, neither of these species have been found at our experimental site.

### Rangeland Management Implications

Our study provides experimental evidence that the effect of warming on forage production and forage quality change seasonally under different moisture conditions. Warming improved the rangeland quality by increasing forage production and there was no change of CP under wet conditions in alpine grassland on the QTP. However, warming might decrease the rangeland quality by decreasing relative importance of grasses but increasing that of forbs and decreasing AGB under dry conditions. Thus, local governments need to take some efficient adaptation strategies, such as setting the grazing intensity according to the grassland forage production and forage quality, and reducing livestock numbers to avoid rangeland degradation, especially in dry regions.

## Conclusion

Experimental warming reduced the average IV of grasses and increased the average IV of forbs under dry conditions, which may indicate a shift in community composition toward fewer grass species and more forb species in alpine systems with few grasses present relative to forbs. A future warmer climate might bring about the increased AGB under wet conditions and cause decreased AGB due to an earlier end of the growing season under dry conditions. Precipitation changes on the QTP will determine whether climate warming is going to benefit rangelands, with drier conditions suppressing grassland productivity, but wetter conditions increasing production while preserving forage quality.

## Author Contributions

FP, QY, and XX proposed the idea and designed the experiment. CLi, FP, CLa, and YC conducted the study. CL and FP wrote the manuscript. QY, XX, CL, and YC revised the manuscript. All authors contributed substantially to revisions and gave final approval for publication.

## Conflict of Interest Statement

The authors declare that the research was conducted in the absence of any commercial or financial relationships that could be construed as a potential conflict of interest.
