# Supplementary material for: Productivity and Quality of Alpine Grassland Vary With Soil Water Availability Under Experimental Warming
Source: Front Plant Sci. 2018 Dec 14;9:1790. doi: 10.3389/fpls.2018.01790 (PMC6302047; doi:10.3389/fpls.2018.01790)
Supplement: Supplementary file 1 [file Table_1.DOCX]

Table S1 Soil pH, CaCO_3_ (g.kg^-2^), soil organic carbon (SOC, g.kg^-2^), total nitrogen (TN, g.kg^-2^), inorganic nitrogen (IN, mg.kg^-2^) and bulk density (BD, g.cm^-3^) in the 0-20 cm, soil temperature (^o^C) and soil moisture (v/v%) in the 0-10 cm, plant coverage (Cov., %) and height (H, cm) in the dry and wet conditions

|  | Soil properties | | | | | | | |  | Plant feature | |
| --- | --- | --- | --- | --- | --- | --- | --- | --- | --- | --- | --- |
|  | PH | CaCO_3_ | SOC | TN | IN | BD | ST | SM |  | Cov. | H |
| Dry | 8.84 ± 0.01 | 5.81 ± 0.23 | 4.91 ± 0.22 | 0.44 ± 0.01 | 8.99 ± 0.38 | 1.25 ± 0.03 | 0.32 ± 0.03 | 6.12 ± 0.02 |  | 65 ± 5% | 6.03 ± 0.64 |
| Wet | 8.33 ± 0.03 | 6.26 ± 0.16 | 10.48 ± 0.95 | 0.88 ± 0.07 | 10.63 ± 0.44 | 1.00 ± 0.05 | -0.16 ± 0.01 | 12.19 ± 0.05 |  | 90 ± 3% | 6.78 ± 1.12 |

Notes: Soil properties were obtained by analyzing soil samples collected in September 2011. Plant height and coverage were also measured in September 2011.
